# Remarks on the diversity of *Hysteromorpha* Lutz 1931 (Digenea, Diplostomidae), with erection of a new species from South America

**DOI:** 10.1007/s11230-025-10224-0

**Published:** 2025-03-29

**Authors:** Tyler J. Achatz, Sean A. Locke, Florencia Arrascaeta, Martin M. Montes, Jorge Barneche, Alan Fecchio, Jeffrey A. Bell, Pablo Oyarzún-Ruiz, Geza T. R. Souza, Ricardo M. Takemoto, Vasyl V. Tkach

**Affiliations:** 1 Department of Natural Sciences, Middle Georgia State University, Macon, GA USA; 2 Department of Biology, University of Puerto Rico at Mayagüez, Mayagüez, PR USA; 3 Centro de Estudios Parasitológicos y Vectores (CEPAVE), Consejo Nacional de Investigaciones Científicas y Técnicas, Universidad Nacional de La Plata (CCT, CONICET-UNLP), La Plata, Buenos Aires Argentina; 4 Department of Ornithology, Academy of Natural Sciences of Drexel University, Philadelphia, PA USA; 5 Department of Biology, University of North Dakota, Grand Forks, ND USA; 6 Departamento de Microbiología, Facultad de Ciencias Biológicas, Universidad de Concepción, Concepción, Chile; 7 Federal Institute of Education, Science and Technology of São Paulo, Salto, São Paulo, Brazil; 8 Postgraduate Program in Ecology of Continental Aquatic Environments (PEA), Limnology, Ichthyology and Aquaculture Research Center (NUPELIA), State University of Maringá, Maringá, Paraná Brazil

## Abstract

*Hysteromorpha* Lutz, 1931 is a small but broadly distributed genus of diplostomoidean digeneans parasitic as adults primarily in cormorants, but also reported from some other fish-eating birds. Their metacercariae were found in a variety of freshwater fishes as second intermediate hosts. Prior to this study, the genus included only 3 nominal species, 2 of them distributed in the Old World and 1 in the New World. We obtained sequences of partial mitochondrial *cox*1 gene and nuclear rDNA operon from new specimens collected in Europe, North and South America and used them for species comparisons and phylogenetic analysis. We also examined morphology of our newly collected specimens as well as museum specimens. Our analysis has demonstrated that at least 2 (likely 3) species of *Hysteromorpha* are distributed in the Americas. *Hysteromorpha* sp. previously sequenced from larval stages, clearly represents a new species named herein *Hysteromorpha ostrowskiae* **n. sp.** Achatz, Locke et Tkach. Morphology of adults of the new species was earlier described in sufficient detail under the name *Hysteromorpha triloba* (Rudolphi, 1819). Our analyses also suggest the presence of another unknown *Hysteromorpha* sp. in North America represented by *cox*1 sequence from southeastern Canada (GenBank JF769473), but no morphological vouchers are available for this species.

**Supplementary Information:**

The online version contains supplementary material available at 10.1007/s11230-025-10224-0.

## Introduction

*Hysteromorpha* Lutz, 1931 is a small genus of diplostomids (Digenea Carus, 1863: Diplostomidae Poirier, 1886) that has a broad geographic distribution (Australia, Asia, Europe, North and South America) (Drago et al., 2011; Dubois, 1970a; González-Acuña et al., 2020; Heneberg et al., 2020; Locke et al., 2018; Sereno-Uribe et al., 2019; Sudarikov, 1960). Although *Hysteromorpha* spp. have been reported from various fish-eating birds, cormorants are their preferred definitive hosts (Dubois, 1970a; Forrester & Spalding, 2003; González-Acuña et al., 2020; Heneberg et al., 2020; Locke et al., 2018; Sereno-Uribe et al., 2019; Sudarikov, 1960). Members of this genus utilize a diversity of freshwater fishes as second intermediate hosts (Dubois, 1970a; Locke et al., 2018; Sudarikov, 1960; Sereno-Uribe et al., 2019).

At present, the genus includes 3 nominal species: *Hysteromorpha triloba* (Rudolphi, 1819), *Hysteromorpha plataleae* Dubinin et Dubinina, 1940 and *Hysteromorpha corti* (Hughes, 1929); *Hysteromorpha triloba* and *H. plataleae* are distributed in the Old World, while *H. corti* is distributed in the New World (Dubois, 1970a; Locke et al., 2018; Sudarikov, 1960). López-Hernández et al. (2019) described cercariae and metacercariae of an additional species-level genetic lineage of *Hysteromorpha* collected in Brazil, but no adults of this lineage have been previously sequenced. Several previous studies have reported adults identified as *H. triloba* in various parts of South America (Drago et al., 2011; González-Acuña et al., 2020; Lutz, 1931).

In the present study, we re-evaluate the diversity of *Hysteromorpha*. We generated sequences of the internal transcribed spacer region (ITS1 + 5.8S + ITS2) and partial large ribosomal subunit gene (28S) of the nuclear ribosomal DNA operon and cytochrome *c* oxidase subunit 1 (*cox*1) mtDNA gene for 3 species of *Hysteromorpha* spp. collected in Europe, North America and South America. Due to its higher variability, *cox*1 sequences were used for distinguishing among species and phylogenetic analysis of the interrelationships among *Hysteromorpha* spp.

## Materials and methods

### Morphological study

Adult specimens of *Hysteromorpha* spp. were collected from intestines of various cormorant species and common redshank *Tringa totanus* (L.) (Scolopacidae Rafinesque) in the USA (Minnesota), Brazil (State of Mato Grosso), Chile (Diguillín Province) and Ukraine (Kherson Oblast). Metacercariae of *Hysteromorpha* spp. were collected from black bullhead catfish *Ameiurus melas* Rafinesque and white sucker *Catostomus commersonii* (Lacépède) in the USA (North Dakota) and peppered cory *Hoplisoma paleatum* (Jenyns) in Argentina (Buenos Aires Province). Live digeneans were heat-killed and preserved in 80% ethanol; specimens for light microscopy were stained with aqueous alum carmine, permanently mounted following Lutz et al. (2017) and examined using an Olympus^©^ BX53 microscope (Olympus America, Pennsylvania, USA) equipped with DIC optics. Type specimens of the new species are deposited by Ostrowski de Núñez (1970) in the Museo Argentino de Ciencias Naturales (MACN). Our voucher specimens are deposited in the collection of the Harold W. Manter Laboratory (HWML), University of Nebraska State Museum, Lincoln, Nebraska and Helminthological Collection of the Museo de La Plata, Argentina (MLP-He) (Table 1).

**Table 1:** Hosts, geographic origin, GenBank and museum accession numbers of *Hysteromorpha* spp. collected in our study and the type material of the new species

*Hysteromorpha* species	Host species	Geographic origin	Museum No.	Accession numbers	
				Ribosomal	*cox*1
*Hysteromorpha ostrowskiae* **n. sp.**	*Hoplisoma paleatum*	Argentina	MLP-He 8170	PQ932488–PQ932491	–
*H. ostrowskiae* **n. sp.**	*Nannopterum brasilianum*	Argentina	MACN 224/14H, 14P, 1–13, 15–24	–	–
*H. ostrowskiae* **n. sp.**	*N. brasilianum*	Brazil	–	PQ932492, PQ932493	PQ901475–PQ901477
*H. ostrowskiae* **n. sp.**	*N. brasilianum*	Chile	HWML 217980	PQ932494, PQ932495	PQ901478, PQ901479
*Hysteromorpha corti*	*Ameiurus melas*	North Dakota, USA	HWML 217978	PQ932496^a^	PQ901480^a^
*H. corti*	*Catostomus commersonii*	North Dakota, USA	–	–	PQ901481
*H. corti*	*Nannopterum auritum*	Minnesota, USA	HWML 217979	PQ932497	PQ901482
*Hysteromorpha triloba* (lineage 1)	*Phalacrocorax carbo*	Kherson Oblast, Ukraine	–	PQ932498	–
*H. triloba* (lineage 1)	*Tringa totanus*	Kherson Oblast, Ukraine	–	PQ932499	PQ901483

Museum abbreviations: HWML, Harold W. Manter Laboratory; MACN, Museo Argentino de Ciencias Naturales; MLP-He, Helminthological Collection of the Museo de La Plata.

^a^Same isolate as GenBank HM114365

### Molecular study

Extraction of genomic DNA from whole specimens was performed according to Tkach & Pawlowski (1999) and amplification by polymerase chain reactions (PCR) occurred in a T100^TM^ thermal cycler (Bio-Rad, California, USA). The forward primer digL2 (5′-AAG CAT ATC ACT AAG CGG-3′) and reverse primer 1500R (5′-GCT ATC CTG AGG GAA ACT TCG-3′) were used to amplify 28S (Tkach et al., 2003). The ITS region was amplified using forward primer ITSF (5′-CGC CCG TCG CTA CTA CCG ATT G-3′) and reverse primer 300R (5′-CAA CTT TCC CTC ACG GTA CTT G-3′) (Littlewood & Olson, 2001; Snyder & Tkach, 2007). The *cox*1 fragments were amplified using forward primers Dipl_Cox_5′ (5′-ACK TTR GAW CAT AAG CG-3′) and JB3 (5′-TTT TTT GGG CAT CCT GAG GTT TAT-3′) and reverse primers Dipl650R (5′-CCA AAR AAY CAR AAY AWR TGY TG-3′), Dipl_Cox_3′ (5′-WAR TGC ATN GGA AAA AAA CA-3′) and JB5 (5′-AGC ACC TAA ACT TAA AAC ATA ATG AAA ATG-3′) (Achatz et al., 2021a; Bowles et al. 1992; Derycke et al., 2005). The PCRs were performed in a total volume of 25 μl using GoTaq G2 DNA Polymerase (Promega, Wisconsin, USA) according to the manufacturer’s instructions and using an annealing temperature of 53 °C for ribosomal amplifications and 45 °C for *cox*1 amplifications.

PCR products were purified using an Illustra ExoProStar PCR clean-up enzymatic kit (Cytiva, Massachusetts, USA). A BrightDye® terminator chemistry kit (Molecular Cloning Laboratories, California, USA) was used to cycle-sequence PCR products. The PCR primers and the newly designed *Hysteromorpha*-specific forward primer Hyst_JB_Int_F (5′-TTG CCC GGG TTT GGA ATG-3′) and reverse primer Hyst_JB_Int_R (5′-GGA TCA CTM ATA CGR AAC CAA CAA CC-3′) were used for sequencing of *cox*1. PCR primers were used for sequencing the nuclear rDNA. The sequencing reactions were purified with a MCLab BigDye® sequencing clean up kit and run on an ABI 3130 automated capillary sequencer (Thermo Fisher Scientific, Massachusetts, USA). Contiguous sequences were assembled using Sequencher ver. 4.2 (GeneCodes Corp., Michigan, USA); new sequences were deposited in GenBank (Table 1).

Two fragments of *cox*1 were used to explore the phylogenetic interrelationships among *Hysteromorpha* spp. Two alignments of *cox*1 were required as previous authors have published non-overlapping sequences of this marker. For instance, Sereno-Uribe et al. (2019) sequenced *Hysteromorpha* isolates from Mexico using the standard barcoding region, while Heneberg et al. (2020) sequenced a downstream *cox*1 fragment of *Hysteromorpha* spp. from Europe. *Neodiplostomum reflexum* Chandler et Rausch, 1947 was used as the outgroup in both alignments. Identical *cox*1 sequences as well as GenBank sequences with clear signs of poor quality (e.g., presence of non-triplet indels) were excluded from analyses and pairwise comparisons.

The first *cox*1 alignment spanned the standard barcoding region established by numerous previous authors (e.g., Folmer et al., 1994; Hebert et al., 2003; Van Steenkiste et al., 2015); this alignment included 6 newly generated and 23 previously published sequences of *Hysteromorpha* spp. The second *cox*1 alignment included a downstream fragment and contained 4 newly generated and 5 previously published sequences of *Hysteromorpha* spp.

MEGA7 (Kumar et al., 2016) identified the general time-reversible model with estimates of invariant sites and gamma distributed among-site variation (GTR + I + G) as the best-fitting nucleotide substitution model for both alignments. The phylogenetic analysis was conducted using Bayesian Inference (BI) as implemented in MrBayes Ver. 3.2.6 software (Ronquist & Huelsenbeck, 2003). The BI analyses were performed using MrBayes software as follows: Markov chain Monte Carlo (MCMC) chains were run for 3,000,000 generations, log-likelihood scores were plotted and only the final 75% of trees were used to produce the consensus tree. This number of generations was considered sufficient because the SD stabilized below 0.01 by the end of the run. Pairwise comparisons of *Hysteromorpha* spp. sequences were performed using MEGA7. In the Results and Discussion the posterior probabilities are expressed as percentages for convenience.

## Results

### Phylogenetic analyses

**Fig. 1:**
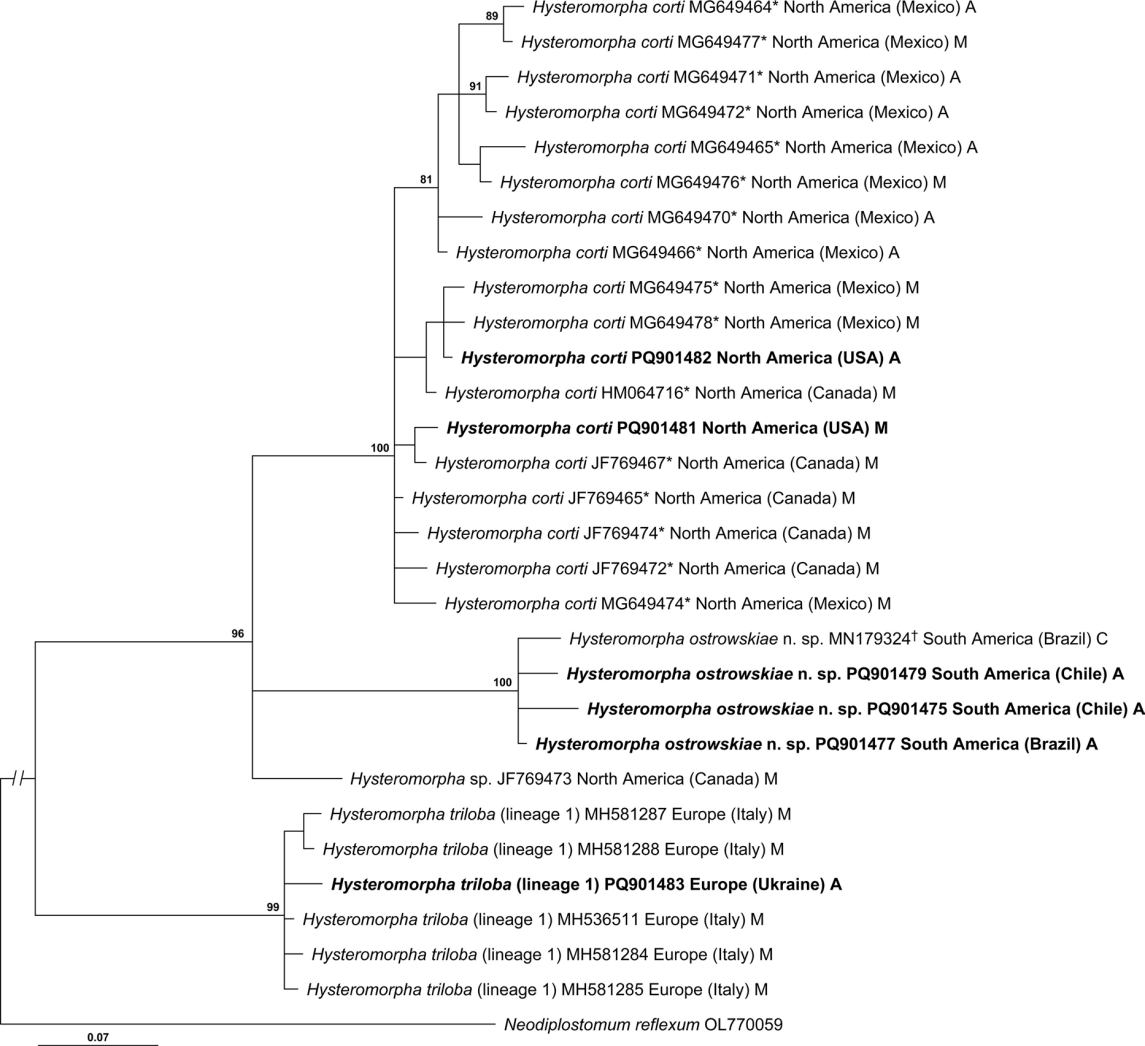
Phylogenetic interrelationships among *Hysteromorpha* spp. based on Bayesian Inference (BI) analysis of cytochrome c oxidase subunit 1 mtDNA gene barcoding region. Bayesian inference posterior probability values lower than 80% are not shown. The new sequences generated in this study are in bold. The scale-bar indicates the number of substitutions per site. Geographic origin and life cycle stage are indicated after GenBank numbers. *H. triloba in GenBank. †*Hysteromorpha* sp. of López-Hernández et al. (2019). Abbreviations for life-cycle stages: A, adult; C, cercaria; M, metacercaria

Upon trimming to the length of the shortest sequence, the alignment of the *cox*1 barcoding region was 429 bp long. The phylogeny resulting from analysis of this alignment was mostly well resolved (Fig. 1). *Hysteromorpha triloba* (lineage 1) isolates from Europe formed a strongly supported clade (99%) as a sister group to a strongly supported clade (96%) of New World *Hysteromorpha* spp. In turn, isolates from the New World split into 3 subclades. The subclades of *H. corti* and *Hysteromorpha ostrowskiae* **n. sp.** (= *Hysteromorpha* sp. of López-Hernández et al. 2019) were 100% supported. It is worth noting that most sequences of *H. corti* included in our analysis were originally deposited in GenBank as *H. triloba* (Sereno-Uribe et al., 2019). The third New-World subclade was represented by a single sequence of *Hysteromorpha* sp. from Canada (deposited in GenBank as *H. triloba*). Importantly, all species-level clades, except for *Hysteromorpha* sp., included data from adult digeneans (Fig. 1).

**Fig. 2:**
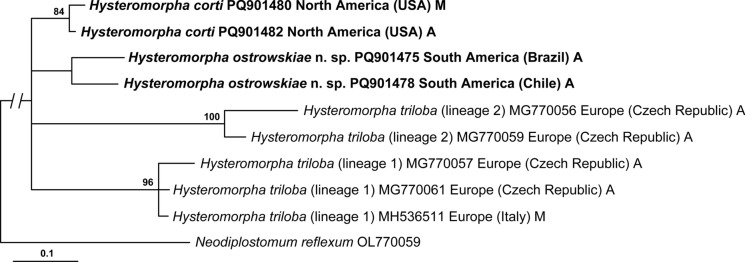
Phylogenetic interrelationships among *Hysteromorpha* spp. based on Bayesian Inference (BI) analysis of the cytochrome c oxidase subunit 1 mtDNA gene fragment downstream from the standard barcoding region. Bayesian inference posterior probability values lower than 80% are not shown. The new sequences generated in this study are in bold. The scale-bar indicates the number of substitutions per site. Geographic origin and life cycle stage after GenBank number. Abbreviations for life cycle stages: A, adult; M, metacercaria

The region of *cox*1 alignment downstream from the barcoding region was 319 bp long upon trimming to shortest length sequence. The phylogeny resulting from the second analysis (Fig. 2) was less resolved compared to the first analysis (Fig. 1). The reduced resolution and support values can be attributed, in part, to the shorter alignment (first analysis—429 bp vs second analysis—319 bp). The second phylogeny (Fig. 2) contained a basal polytomy with 4 clades. The clades containing *H. corti* from North America (84% supported) and *H. ostrowskiae* **n. sp.** from South America (below 80% support) had much weaker support compared to the first analysis. Two clades of *H. triloba* were recovered, referred to on the tree as lineages 1 and 2 (Fig. 2); both *H. triloba* lineages exhibited strong branch support values (96% and 100%, respectively).

The phylogenetic position of *Hysteromorpha* within the Diplostomoidea based on ribosomal loci has been analyzed in several previous publications (e.g., Achatz et al., 2023, 2024; Blasco-Costa & Locke, 2017; Locke et al., 2018; López-Hernández et al., 2019; Sereno-Uribe et al., 2019); therefore, we are not discussing it here.

### Pairwise comparisons

The sequences of the barcoding region of *cox*1 demonstrated interspecific divergence (3.7–9.6%, Table S1) similar to what has been observed in other diplostomoidean genera (Achatz et al., 2021b; Locke et al., 2018; and references therein; Gudla et al. 2023). *Hysteromorpha triloba* (lineage 1) + *H. corti* and *H. triloba* + *H. ostrowskiae* **n. sp.** were the most divergent pairs in the barcoding region (9.6%), while *H. corti* + *Hysteromorpha* sp. had the lowest divergence (3.7–5.6%). The intraspecific variation in the *cox*1 barcoding region was much lower than interspecific divergence: 0–2.6% in *H. corti*; 0.7–1.9% in *H. ostrowskiae* **n. sp.** and 0.2–1.2% in *H. triloba* (lineage 1).

The pairwise comparisons of the *cox*1 fragment downstream from the barcoding region showed levels of divergence (5.3–11.9%, Table 2) overall similar to those in the barcoding region. *Hysteromorpha triloba* (lineage 2) + *H. ostrowskiae* **n. sp.** were the most divergent pair of species in this region (11.9%) while *H. ostrowskiae* **n. sp.** + *H. corti* were the least divergent pair (5.3%). Intraspecific variation in the downstream *cox*1 fragment was notably different: *H. corti*—0.6%; *H. ostrowskiae* **n. sp.**—4.1%; *H. triloba* (lineage 1)—up to 1.9%; *H. triloba* (lineage 2)—3.8%.

**Table 2:** Pairwise comparisons of *Hysteromorpha* spp. *cox*1 sequences of based on downstream alignment (319 bp long)

		1.	2.	3.	4.	5.	6.	7.	8.	9.
1.	*Hysteromorpha corti* PQ901480	–	0.6%	6.0%	6.0%	8.5%	8.8%	7.8%	10.0%	8.8%
2.	*H. corti* PQ901482	2	–	5.3%	5.3%	7.8%	8.2%	7.2%	9.4%	8.2%
3.	*Hysteromorpha ostrowskiae* **n. sp.** PQ901478	19	17	–	4.1%	6.6%	7.5%	6.6%	11.0%	9.7%
4.	*H. ostrowskiae* **n. sp.** PQ901475	19	17	13	–	7.2%	8.2%	7.2%	11.9%	10.7%
5.	*Hysteromorpha triloba* (lineage 1) MH536511	27	25	21	23	–	1.9%	0.6%	11.6%	10.3%
6.	*H. triloba* (lineage 1) MG770057	28	26	24	26	6	–	1.9%	11.6%	9.7%
7.	*H. triloba* (lineage 1) MG770061	25	23	21	23	2	6	–	11.0%	10.3%
8.	*H. triloba* (lineage 2) MG770056	32	30	35	38	37	37	35	–	3.8%
9.	*H. triloba* (lineage 2) MG770059	28	26	31	34	33	31	33	12	–

Percentage differences given are above the diagonal and number of base pair differences are given below the diagonal. GenBank numbers provided after species name

The 28S sequences of *Hysteromorpha* spp. exhibited no interspecific variation except for 2 unresolved sites (all cytosine or thymine) in 3 *H. corti* isolates (GenBank accessions OQ211111, PQ932496 and PQ932497). Interestingly, the newly generated ITS region sequences from North and South America were completely identical, except for an *H. corti* isolate from the USA (PQ932496) and an *H. ostrowskiae* **n. sp.** from Brazil (PQ932493) that had 1 unresolved site each (cytosine or thymine and adenine or thymine, respectively). However, when compared with previously published sequences in GenBank, some variation was observed between *H. corti* collected in the USA and Mexico (0–0.2%). The ITS region sequences generated in the present study and those of *H. triloba* (lineage 1) of Locke et al. (2018) were identical; these sequences had a single nucleotide difference from the North American species. The ITS sequences of *H. triloba* (lineage 1) available in GenBank had up to 2 variable nucleotides (0.3%), and ITS sequences of *Hysteromorpha triloba* (lineage 2) available in GenBank had 1 nucleotide difference (0.2%). The 2 *H. triloba* lineages differed between zero and 3 nucleotides (0.5%) in ITS region sequences in GenBank as well as our new sequence. It is worth noting that previous studies reported 0.3% intraspecific variation in *Hysteromorpha* spp. sequences of the ITS region (Locke et al., 2018; Sereno-Uribe et al., 2019).

## Discussion

### Species diversity

Rudolphi (1819) originally described *H. triloba* (as *Distoma trilobum*) based on European material. Lutz (1931) erected *Hysteromorpha* for *H. triloba* based on specimens from Brazil, which were likely identified incorrectly based on current knowledge. Prior to the present study, the genus included 3 nominal species: *H. triloba* (type-species), *H. plataleae* and *H. corti* (Dubois, 1970a; Locke et al., 2018). Prior to Locke et al. (2018), *H. triloba* was considered the only member of the genus in North and South America based on adult morphology (Dubois, 1970a; Hugghins, 1954; Lutz, 1931; Ostrowski de Núñez, 1970). López-Hernández et al. (2019) collected and described immature stages of *Hysteromorpha* sp. in Brazil. Our newly collected adult specimens from Brazil and Chile, along with metacercariae from Argentina, are conspecific with the specimens described by López-Hernández et al. (2019) from Brazil based on genetic comparisons (Table 2, S1; Fig. 1). Our analyses also suggest the presence of another unknown *Hysteromorpha* sp. in North America represented by the sequence from southeastern Canada (JF769473).

Locke et al. (2018) noted the lack of consistent morphological differences between adult stages of *H. triloba* (lineage 1) and *H. corti*. Unfortunately, we also have failed to find consistent morphological differences between adult specimens of these species, as well as the *Hysteromorpha* sp. of López-Hernández et al. (2019). Despite the similarity of adult stages, metacercariae of these species do differ. *Hysteromorpha triloba* has an oral sucker, pharynx and esophagus that are generally larger than in *H. corti* (Locke et al., 2018). Similarly, *Hysteromorpha* sp. of López-Hernández et al. (2019) generally has a smaller ventral sucker and longer holdfast organ compared to *H. triloba* and *H. corti* (López-Hernández et al., 2019). While these morphological differences are minor, the level of divergence among *cox*1 sequences among these species (6.1–9.6% in the barcoding region; Table S1) strongly suggests that they represent separate species. Based on the available data, *H. corti* appears to be limited to North America (Locke et al. 2018; present study), while *H. triloba* is likely restricted to Eurasia and *H. ostrowskiae* **n. sp.** (= *Hysteromorpha* sp. of López-Hernández et al. (2019)) is only known from South America.

Ostrowski de Núñez (1970) provided detailed description of specimens identified as *H. triloba* collected from neotropic cormorant *Nannopterum brasilianum* (Gmelin) (referred to as *Phalacrocorax b. brasilianus*) in Argentina. Since it is now clear that the South American form/genetic lineage represents a separate species, we name it *H. ostrowskiae* **n. sp.** Achatz, Locke et Tkach. We do not provide a description of our specimens as the description provided by Ostrowski de Núñez (1970) was of sufficient quality and consistent with the morphology of our specimens. Descriptions of the cercariae and metacercariae of this species were provided by López-Hernández et al. (2019). Taxonomic summary for the new species is provided below.

#### Taxonomic summary

*Type host*: *Nannopterum brasilianum* (Gmelin) (Suliformes: Phalacrocoracidae).

*Site of infection*: Small intestine.

*Type locality*: Buenos Aires Province and Córdoba Province, Argentina.

*Type material*: The type-series is deposited in the Helminthological Collection of the Museo Argentino de Ciencias Naturales in Buenos Aires. Holotype: MACN-Pa 224/14H. Paratypes (5 specimens on same slide as holotype): MACN-PA224/14P. Vouchers: MACN-PA224/1–13, 15–24 (224/20–24 with histological sections).

*Representative DNA sequences*: ribosomal (PQ932488–PQ932495); *cox*1 (PQ901475–PQ901479).

*Zoobank registration*: urn:lsid:zoobank.org:act:13507F21-BD68-4599-A7C3-561AC2E1382A.

*Etymology*: The new species is named in memory of Dr. Margarita C. Ostrowski de Nuñez and in recognition of her contributions to the knowledge of Neotropical digeneans.

There are a number of South American records of *H. triloba* in the literature (Fernandes et al., 2015; Table 3). However, these reports were not supported by molecular data and almost none contained illustrations. Although most of them likely represented *H. ostrowskiae* **n. sp.**, we cannot be certain that all belong to this species. First, distribution of the double-crested cormorant *Nannopterum auritum* (widely distributed in North America) and Neotropic cormorant (widely distributed in South America) have a significant area of overlap in northern part of the Neotropics and southern Nearctic, a historical exchange with helminths between the two host species with subsequent geographic radiation is feasible. Second, there is a possibility of regional speciation that has not been uncovered yet using molecular tools.

**Table 3.** Reports of *Hysteromorpha* from South America

Country	Host	Life stage	Reference
Argentina	*Hoplisoma paleatum*	Metacercaria	Present study
	*Nannopterum brasilianum*	Adult	Ostrowski de Núñez (1970); Drago et al. (2011)
Brazil	*Biomphalaria straminea*	Cercaria	López-Hernández et al. (2019)
	*N. brasilianum*	Adult	Lutz (1931); Monteiro et al. (2011); Carvalho et al. (2024); Present study
Chile	*N. brasilianum*	Adult	González-Acuña et al. (2020); Present study
Venezuela:	*N. brasilianum*	Adult	Dubois (1970b); Noronha et al. (2009)

Szidat (1969) described a metacercaria *Tetracotyle loricariae* Szidat, 1969 from *Loricaria anus* Valenciennes (currently *Loricariichthys anus*) in Argentina. Sudarikov (1974) transferred that metacercaria into *Hysteromorpha* as *Hysteromorpha loricariae* (Szidat, 1969). However, in the same paragraph Sudarikov (1974) wrote: “Superficial description of this species does not allow to make a definitive conclusion about its position in the system of strigeates”. In other words, his nomenclatural act was unwarranted. In our opinion, there is no ground to consider *H. loricariae* and *H. ostrowskiae* **n. sp.** conspecific. The illustration and description of the metacercaria by Szidat (1969) was so superficial that several diplostomoidean taxa could potentially fit into that description. Importantly, Szidat’s drawing clearly lacks pseudosuckers, which is a characteristic feature of *Hysteromorpha*. Therefore, consideration of the two forms conspecific would be unwarranted.

Heneberg et al. (2020) sequenced several adult *H. triloba* from the great cormorant *Phalacrocorax carbo* (L.) in the Czech Republic. Our phylogenetic analysis (Fig. 2) and pairwise comparisons (Table 2) of the fragment downstream from the *cox*1 barcoding region suggest that the Czech materials represent 2 species-level lineages, both identified as *H. triloba*. The first lineage is conspecific with our European isolates. These European lineages differ by 9.7–11.6%, which far exceeds minimal levels of interspecific variation observed in diplostomoideans (3.4–22.3% in *cox*1; e.g., Achatz et al., 2021b; Locke et al., 2018; Tkach et al., 2020).

While most reports of *Hysteromorpha* spp. have been from cormorants, *H. plataleae* was described from Eurasian spoonbill *Platalea leucorodia* L. (Threskiornithidae Richmond) and reported from red-naped ibis *Pseudibis papillosa* (Temminck) (Threskiornithidae) (Dubinin & Dubinina, 1940; Odening, 1962). It has never been reported from cormorants (Dubois, 1970a). It is possible that 1 of the 2 *H. triloba* lineages represents *H. plataleae* or a potentially undescribed species. It is unclear what range of avian hosts may be used by any *Hysteromorpha* spp. Recently, Baumann et al. (2024) reported *H. corti* from a brown booby *Sula leucogaster* (Boddaert), another suliform. We collected our *H. triloba* (lineage 1) specimens from great cormorant and common redshank, a scolopacid, from Ukraine. As far as we are aware, this is the first report of any *Hysteromorpha* sp. from a scolopacid. Collection of new high-quality adults and metacercariae of both *H. triloba* lineages as well as the *Hysteromorpha* sp. remain needed to address the question of identities and taxonomic status of these digeneans.

## Supplementary Information

Below is the link to the electronic supplementary material.

Supplementary file1 (XLSX 20 KB)

## Data Availability

No datasets were generated or analysed during the current study.
